# Prenatal diagnosis and pregnancy outcome of acardiac amorphus twin

**DOI:** 10.11604/pamj.2022.42.5.34633

**Published:** 2022-05-05

**Authors:** Mahdi Farhati, Abir Karoui

**Affiliations:** 1 Tunis Maternity and Neonatology Center, Tunis, Tunisia

**Keywords:** Twin reversed arterial perfusion, ultrasound, prenatal diagnosis

## Image in medicine

We report a case of a 35 years old primigravida patient, referred at 17 weeks of gestation to our consultation for suspicion of a twin pregnancy with vanishing twin. Ultrasound exam showed one fetus with appropriate for gestational age dimensions next to an amorphus mass of 59 mm (A,B). A reversal in arterial flow was noted on a Doppler study, coming from the apparently normal fetus to the mass, and the diagnosis of acardiac twin was made. In view of the absence of negative ultrasound signs (umbilical artery PI ratio, umbilical artery RI differences, weight ratio between acardiac mass and pump twin, heart function of pump twin and amniotic fluid volume) we decided to follow the pregnancy by fortnightly ultrasound checks. At 29 weeks of gestation, a bilateral ventriculomegaly was discovered at the cephalic level without any signs of heart decompensation. A fetal brain MRI was performed at 32 weeks of gestation and showed porencephaly and schizencephaly lesions related to anoxischemic and hemorrhagic sequelae. Intra-uterine fetal death occurred at 32 weeks of gestation. The examination after evacuation showed a stillborn of 1800g of normal morphology, an amorphous acardiac fetus with an outline of a limb weighing 200g (C).

**Figure 1:**
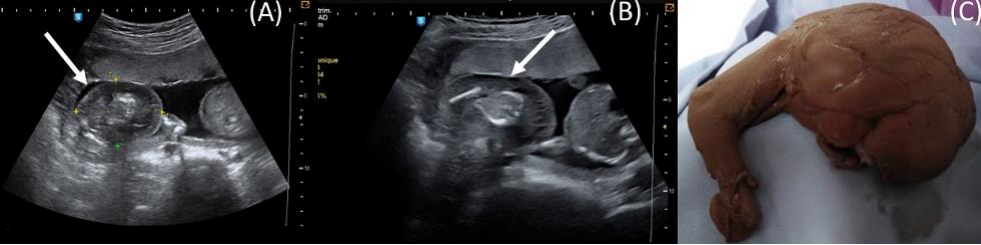
acardiac amorphus twin

